# The effect of N-ethyl-N-hydroxyethyl perfluorooctanoamide on wettability alteration of shale reservoir

**DOI:** 10.1038/s41598-018-25100-9

**Published:** 2018-05-02

**Authors:** Yongfei Li, Yanling Wang, Kun Wang, Foster Gomado, Gangxiao Wang, Longhao Tang, Xufeng Rong

**Affiliations:** College of Petroleum Engineering, China University of Petroleum (East China), Qingdao, 266580 China

## Abstract

The wettability of the formation is critical for the flow back of the fracturing fluid and can further affect the gas production. So it is very necessary to study the wettability of shale reservoir. Here, a novel fluorocarbon surfactant, N-ethyl-N-hydroxyethyl perfluorooctanoamide, was synthesized and characterized by different methods. the contact angles of water and n-decane on the shale increased from 36° and 0° to 121° and 105°, respectively, after treated by N-ethyl-N-hydroxyethyl perfluorooctanoamide (0.5 wt.%). The surface free energy reduced from 72 mN/m to 7.4 mN/m. The results agreed with that of imbibition and capillary tube rise test. Additionally, the analysis of scanning electron microscope (SEM) and energy dispersive spectroscopy (EDS) showed that the roughness of shale surface remarkably increased. These results fully proved that the shale wettability is changed to super gas-wetting. Besides, the thermal analysis revealed that the novel fluorocarbon surfactant has good thermal stability. This indicates that it can be better applied to reservoir modifications at higher temperatures.

## Introduction

Shale gas resources are relatively abundant in some countries or regions around the world. And it is more environmentally friendly than other fossil fuels^1,2^. Therefore, it can effectively deal with environmental pollution and promote economic growth. Especially in China^3^, shale gas can also be used as a supplement to traditional energy to address the serious shortage of energy. However, it is typically formed in low-permeability or nanoscale pores media^4^. Because of its unique pore feature, it is not easy to capture from the reservoir without extra driving force^5^. The improvement of hydraulic fracturing technology has promoted the rapid development of shale gas^6–8^. Although the technology is very significant for the increase in production, its typical characteristics are high liquid volume and large displacement. So fracturing fluid flowback plays a vital role in shale gas development. Its purpose is to reduce the reflow of the proppant and to maintain the reservoir cracks with a strong ability for diversion.

In recent years, the research on the theoretical system of “gas-wetting” has attracted much attention^9^. If the wettability of the shale reservoir is altered to gas-wetting, the fracturing fluid flowback can be effectively improved. Wettability refers to a phenomenon in which a fluid is replaced by another fluid on a solid surface^10^. Conventional wettability is divided into oil-wetting and water-wetting. The so-called “gas-wetting” means that it is hydrophobic and oleophobic^11^. Moreover, wettability is a crucial factor to control the distribution of fluid in porous media, which plays an important role in the seepage and recovery of gas reservoir^12^. Additionally, it is an indispensable physical parameter for the evaluation, modification and dynamic analysis of gas reservoirs. Thus, the research on the gas-wetting has opened up a new field in petroleum and chemical industry. Tang and Firoozabadi^13^ investigated that the relative permeability of the Berea and chalk samples can increase significantly by using two fluoropolymers, FC-722 and FC-759. Stanley and Feng *et al*.^14^ reported that wettability of tightly porous media have been perennially altered to intermediate gas-wetting after treatment with novel fluoropolymers. However, the above-mentioned studies have shown that only fluoropolymer was evaluated for the ability to alter wettability. Due to the large molecular weight of the polymer, it is difficult to degrade naturally, resulting in contamination of the formation and water resources. At the same time, polymers tend to plug the pores of the formation, causing a loss of oil and gas production. Therefore, some researchers have proposed to change the wettability of the reservoir surface by non-polymers. Aminnaji^15^ investigated that a nanofluid can change the rock wettability to neutral gas wetness within 24 h. Esmaeilzadeh^16^ reported on a non-polymer material prepared by the gel method, which has good hydrophobic and oleophobic properties and can alter the surface of the reservoir from strong liquid wetness to gas wetness. Jin and Wang^17^ altered the wettability of gas condensate reservoirs from liquid-wet to gas-wet by non-polymeric fluorosurfactant, which can be used to overcome the difficulties of the liquid lock effects and further significantly improve the gas recovery. Therefore, gas- wetting surface can be obtained through the non-polymeric materials.

The focus of the work is to synthesize a small molecule fluorosurfactant with low surface free energy, which can alter the shale wettability to super gas-wetting in shale gas reservoirs. Furthermore, it can more remarkably improve the flowback rate of the fracturing fluid and the gas recovery.

## Results and Discussion

### Structure

**Figure 1:**
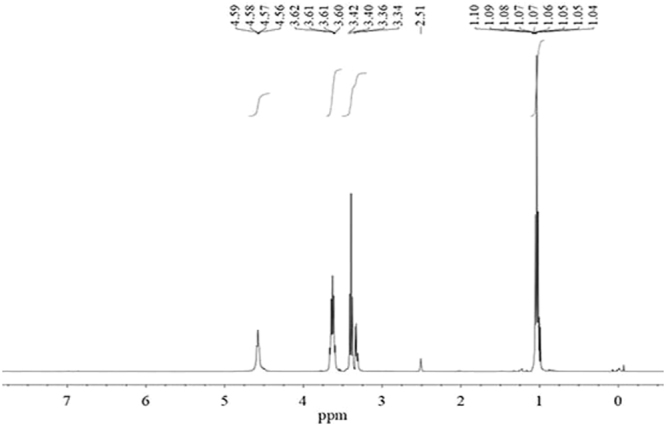
The ^1^HNMR spectrum of the novel fluorocarbon surfactant.

The novel fluorocarbon surfactant was characterized by ^1^HNMR. Figure 1 exhibits its spectrum: ^1^HNMR (400 MHz, DMSO-*d*_6_), δ: 4.58 (t, *J* = 6.5 Hz, 1H), 3.61 (d, *J* = 5.0 Hz, 2H), 3.42–3.38 (m, 4 H), 1.07 (d, *J* = 7.1 Hz, 3H). The peak at δ 4.58 for H corresponds to the -OH group. The peak at δ 3.61 is generated by the methylene attached to the hydroxyl group. The presence of multiple peaks at δ 3.42-3.34 corresponds to the two methylene adjacent to the N atom. The peak of terminal methyl appears at δ 1.07. The peak at δ 2.51 is due to the solvent (DMSO). The result agrees with the structural proportions of the synthesized compound^18,19^.

**Figure 2:**
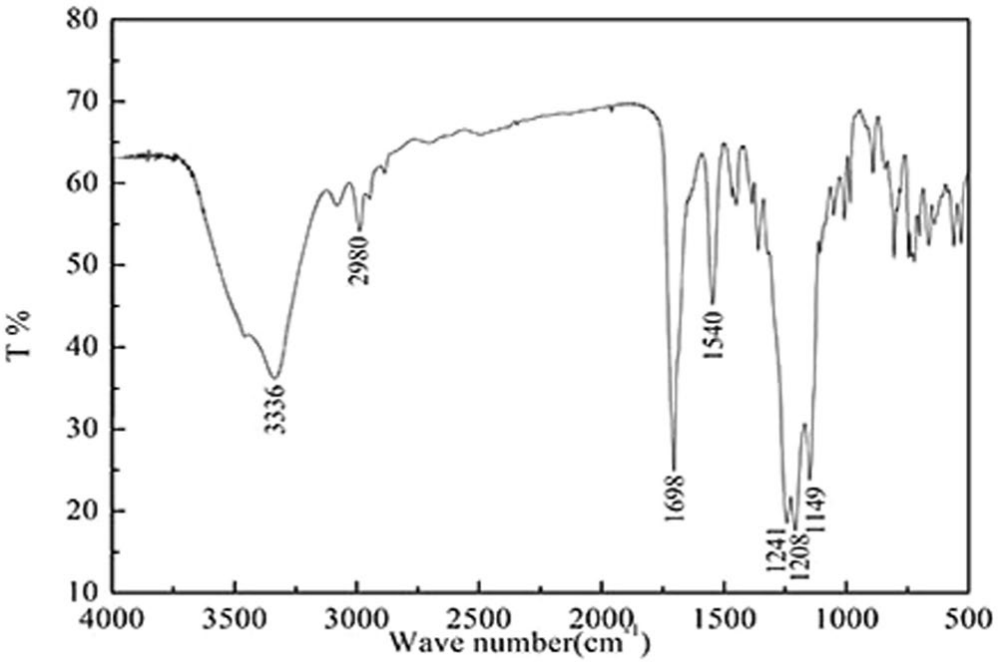
FTIR spectra of the novel fluorocarbon surfactant.

Furthermore, the structure of the product was confirmed by infrared spectroscopy and shown in Fig. 2. The characteristic absorption peak of -OH appears at 3336 cm^−1^. The peaks at 2980 cm^−1^ and the vicinity correspond to the characteristics of absorption of the -CH_3_ and -CH_2_-. The deformation vibration of -CH_2_- occurs in 1540 cm^−1^. At 1698 cm^−1^ is the stretching vibration peak. Additionally, the characteristics absorption of C=O-NH group at 3300 cm^−1^–3100 cm^−1^ disappear. So the monomers have fully reacted. Besides, the absorption peaks of C-F group occur in 1241 cm^−1^, 1208 cm^−1^ and 1149 cm^−1^ respectively. It is consistent with the nuclear magnetic analysis. These results above reveal that the product has been successfully synthesized.

### EDS and SEM

**Figure 3:**
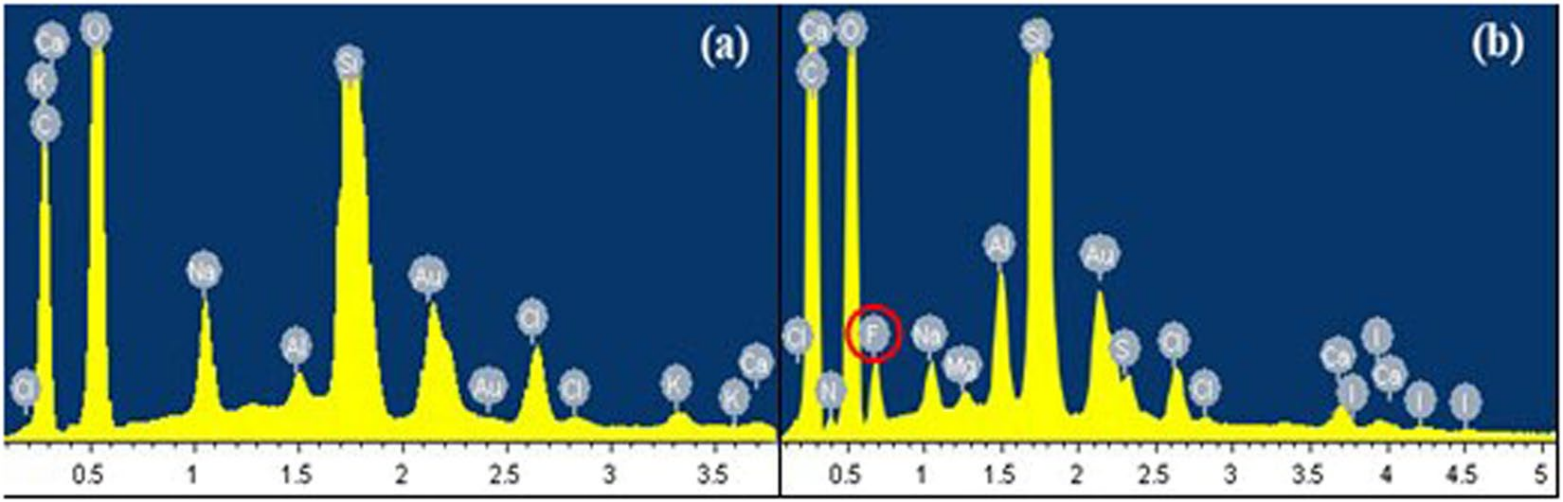
EDS of the shale surface: (**a**) untreated rock, (**b**) treated.

**Figure 4:**
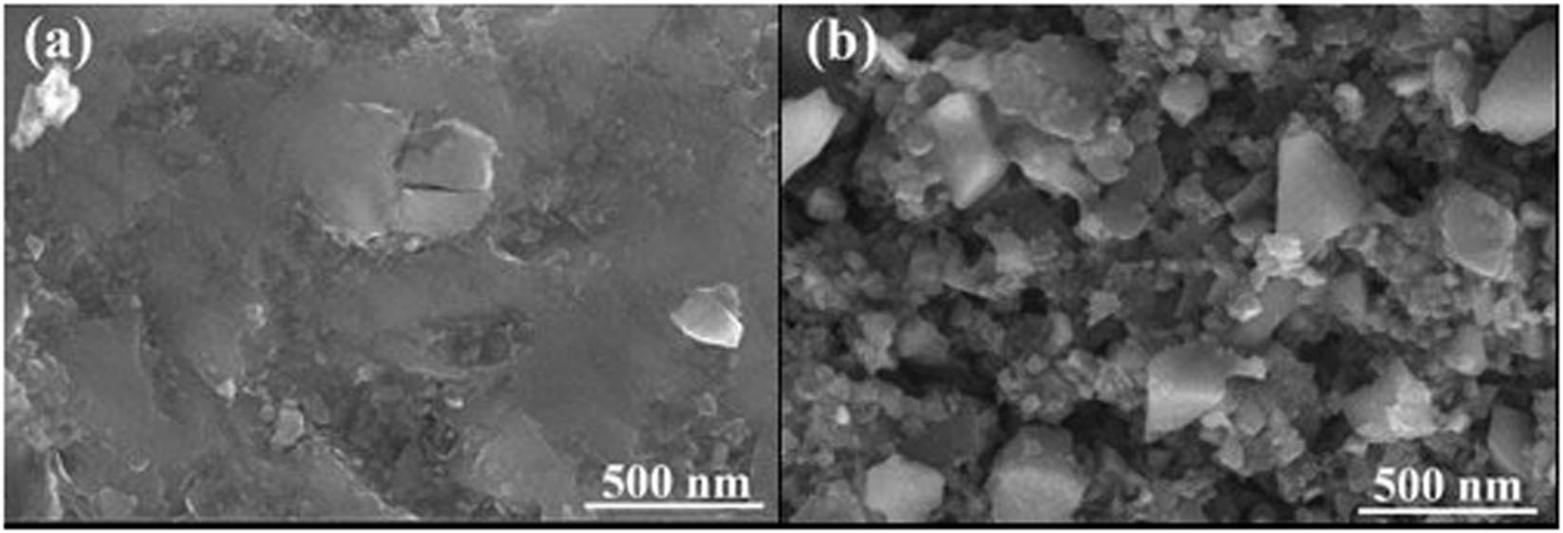
SEM of the shale surface: (**a**) untreated rock, (**b**) treated.

Figures 3 and 4 display the elemental changes and microscopic morphology of the shale surface before and after it was modified by the fluorocarbon surfactant. As shown in Fig. 3(a), The fluorine was not found in the untreated shale and includes other elements such as calcium, potassium, oxygen and silicon. It can be seen from Fig. 3(b) that the fluorine is detected on the treated shale sample. Thus, the fluorine can be sufficiently adsorbed on the surface of the shale, which is the primary cause to achieve gas-wetting reservoir.

Figure 4(a) shows that almost no material is covered on the surface of the original shale and its surface is relatively smooth, which can promote the expansion of liquid on the shale surface. However, it can be clearly seen from Fig. 4(b) that the surface morphology of the shale has been significantly changed after treatment with the fluorocarbon surfactant. The surface of the shale adsorbs a large number of chemical molecules and forms a tight adsorption layer, which effectively improves the roughness of the shale surface. In addition, the surface free energy can be significantly reduced after treatment. Thus, the wettability of the shale surface can be altered from liquid-wetting to gas-wetting.

### Thermal analysis

**Figure 5:**
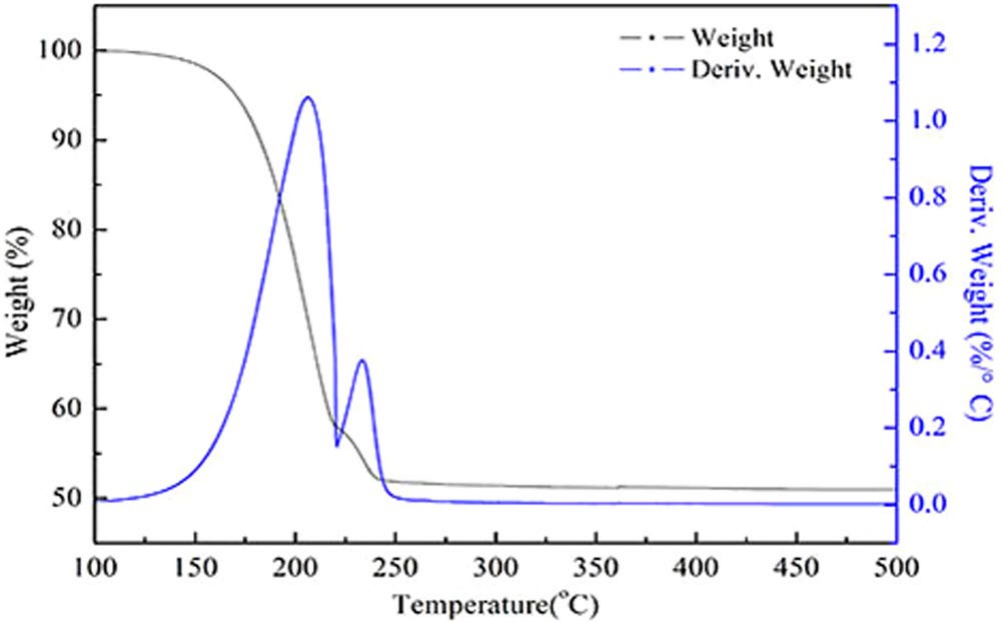
The thermal analysis curves of the novel fluorocarbon surfactant.

The thermogravimetric analysis reveals the stability of the novel fluorocarbon surfactant with increasing temperature. As seen from in Fig. 5. There are three phases of weightlessness in the thermal analysis curve. The first stage is very little weight loss before 150 °C, which may be the evaporation of the residual solution. In the second, there was a significant increase of weight loss between 150 °C and 245 °C, with the maximum weight loss rate peaking at about 1.061%/°C at 205 °C. And the reaction interval is relatively narrow. It shows that the compound has been thermally decomposed at this stage. Above 205 °C, the weight remains nearly unchanged. The Thermogravimetric result reveals that the decomposition of the novel fluorocarbon surfactant can proceed under relatively high-temperature^20,21^.

### Contact angle measurement

**Figure 6:**
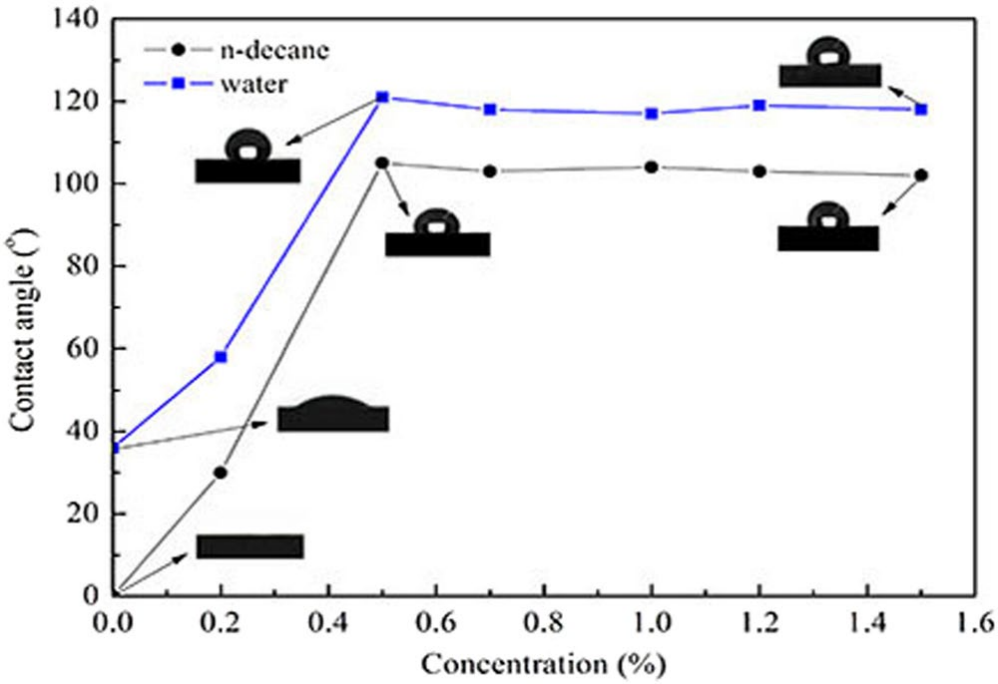
The contact angles of water and n-decane on shale surfaces.

The contact angles of droplets on the shale were measured and shown in Fig. 6. The shale is strong liquid wetness when it is not treated. But after treated with the novel fluorocarbon surfactant, the shale wettability is changed to preferential gas wetness. When the concentration of the novel fluorocarbon surfactant is 0.2 wt.%, the contact angle of water is changed from 36° to 58°, while that of n-decane increase from 0° to 30°. With the increase of novel fluorocarbon surfactant concentration, the contact angles of water and n-decane increase continuously. When the concentration is 0.5%, the contact angles reached a maximum to 121° and 105°, respectively. The results show that the shale is altered from liquid wetness to gas wetness. Following the respective contact angles of water and n-decane remain about 120° and 105° as increases in concentration.

This phenomenon may be explained as following: when the concentration is low, a small amount of molecules is adsorbed on the shale surface and they are easily replaced by other molecules. When the concentration is high, a great quantity of molecules adsorbs on the shale surface and form a molecular adsorption layer^22,23^. As a result of this, the wettability of the shale is changed to super gas wetness. As the concentration continues to increase, the adsorption capacity of shale becomes saturated and stable. Therefore, the contact angle of water or n-decane remains essentially constant^24^.

### The surface free energy

**Figure 7:**
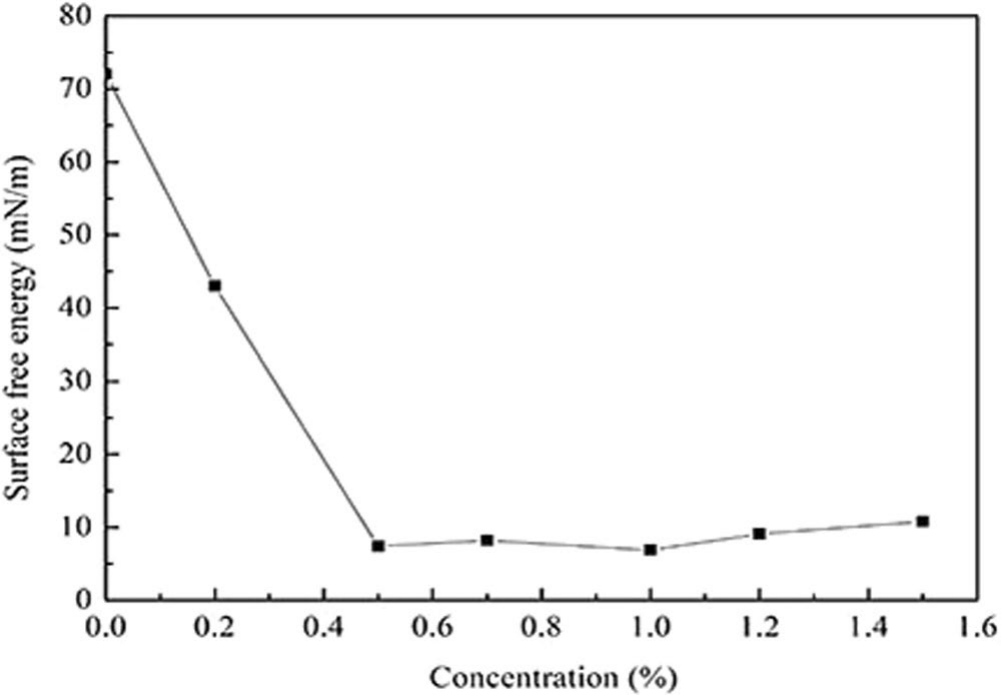
The surface free energy of the shale.

Figure 7 illustrates the surface free energy of the shale before and after treatment with the novel fluorocarbon surfactant. It is clear that the surface free energy reduced from 72 mN/m to 7.4 mN/m after treatment with a concentration of 0.5 wt.%. As the concentration increases, the surface free energy is almost constant. However, when the concentration is greater than 1.0%, it tends to increase gradually. It may be due to the fact that the polar part of the fluorocarbon surfactant adsorbs to the shale, while the non-polar part is exposed on the surface and forms a gas-wetting molecular layer. Therefore, the wettability of the shale surface was changed. However, when the concentration exceeds the optimum amount, the molecular chains may become entangled with each other, resulting in some non-polar portions being shielded. So that a portion of the nonpolar segments is locked. Therefore, the surface free energy has a tendency to rebound^25^.

### Imbibition

**Figure 8:**
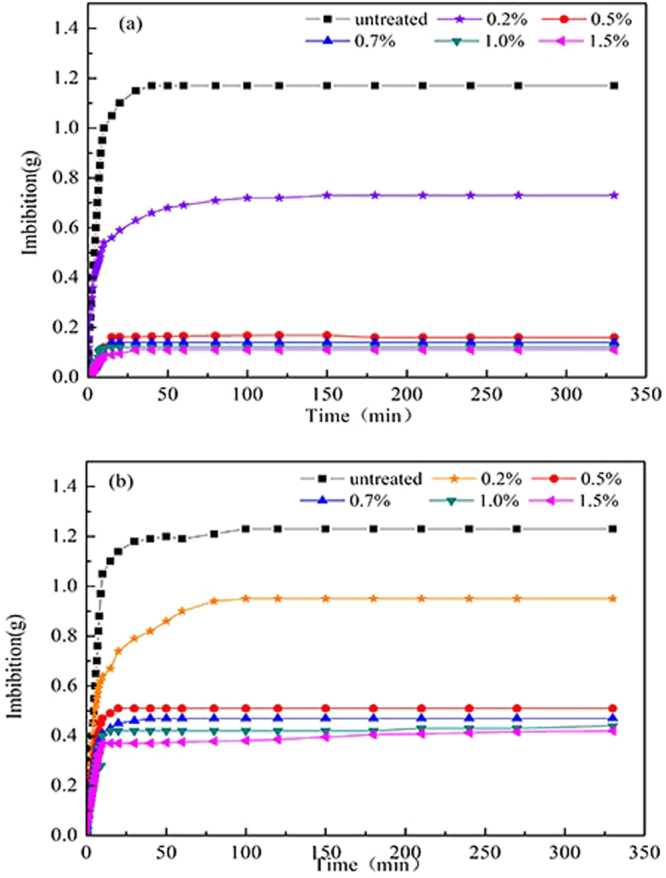
Imbibition of liquid in the shale: (**a**) water; (**b**) n-decane.

Imbibition test can effectively reflect the wettability of shale. Figure 8(a) illustrates that the volume of water imbibed into the shale was 1.17 g without the chemical treatment, which sharply decreased to 0.16 g after the treatment with 0.5 wt.%. It demonstates that the shale surface has strong hydrophobic properties after altering the wettability with the novel fluorocarbon surfactant. Figure 8(b) depicts the n-decane imbibition in untreated and treated shale. The amount of imbibed shale decreased significantly from 1.23 g to 0.51 g after treatment, which indicates that shale surface become oleophobic. The decrease in the inhalation volume of water and n-decane proves that the shale wettability has been changed to super gas wetness, which is consistent the results of the contact angle measurement and surface free energy^23^.

### Capillary tube rise test

**Figure 9:**
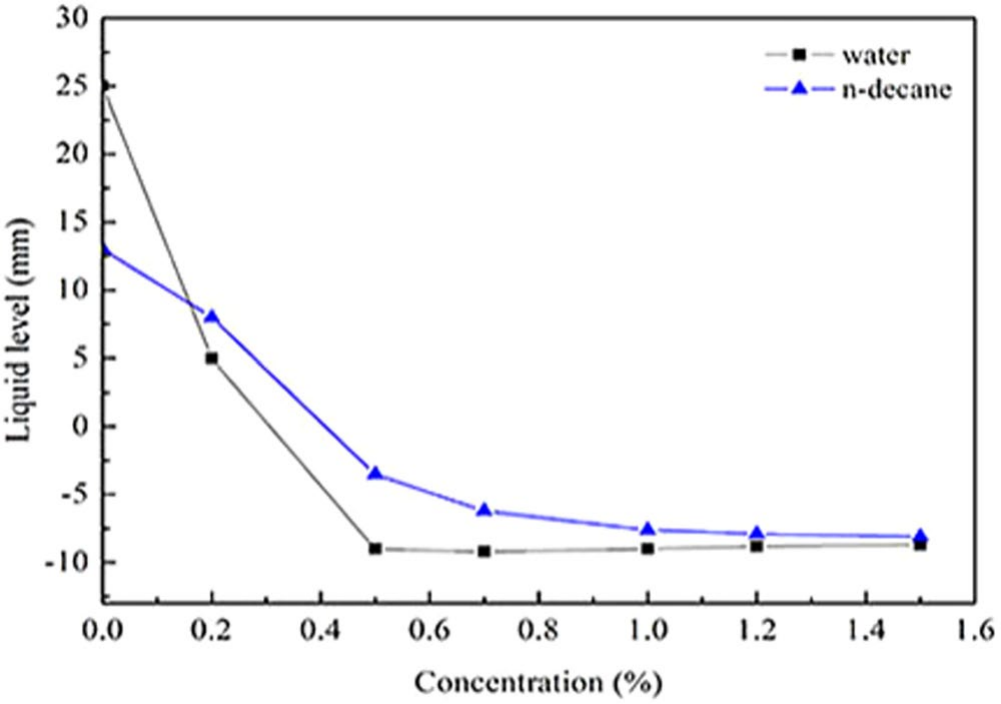
The height of the water and n-decane suction capillary.

The capillary tube rise experiment was used to further demonstrate the wettability alteration of the shale. Figure 9 summarizes that the height of the water and n-decane suction capillary as a function of concentration. It is very obvious that the height of water was reduced from 25 mm to −9 mm as the concentration of fluorocarbon surfactant increased, While the n-decane level was confirmed to have a similar trend with the former. Therefore, it is sufficient to indicate that the wettability of the capillary inner wall has been changed to gas-wetting. The result of glass capillary rise is consistent with that of spontaneous imbibition. Therefore, the wettability of the shale is changed to gas-wetting state in the shale gas reservoir, which can significantly increase the flowback rate of the working fluid and maintain a high production of the reservoir.

## Conclusions

Reservoir wettability plays a vital role in the production of shale gas. In this work, a novel fluorocarbon surfactant which can be applied to alter reservoir wettability was synthesized and it has good temperature resistance before 150 ^o^C. The wettability of shale surface was evaluated by different method. The results showed that the contact angles of water and n-decane were changed from 36° and 0° to 121° and 105°, respectively, after treatment with 0.5 wt.% of the synthesized chemical. The surface free energy reduced from 72 m/Nm to 7.4 m/Nm. The results are consistent with that of imbibition and capillary tube rise experiment. In addition, the analysis of SEM and EDS illustrate that the roughness of the shale surface has been increased significantly. These results fully reveal that the shale wettability altered from liquid wetness to super gas wetness.

## Experimental

### Materials

Perfluorooctanoic acid and Thionyl chloride were purchased from Aladdin Industrial Corporation. Isopropyl ether, potassium hydroxide and anhydrous ethanol were provided by Nanjing Chemical Reagent. Ethylamine and triethylamine were supplied by Shandong Chemical Reagents Co., Ltd. 2-chloroethanol was purchased from Tianjin Kemiou Chemical Reagent Co., Ltd. The shale samples were provided by Shengli Oilfield.

### Synthesis

**Figure 10:**
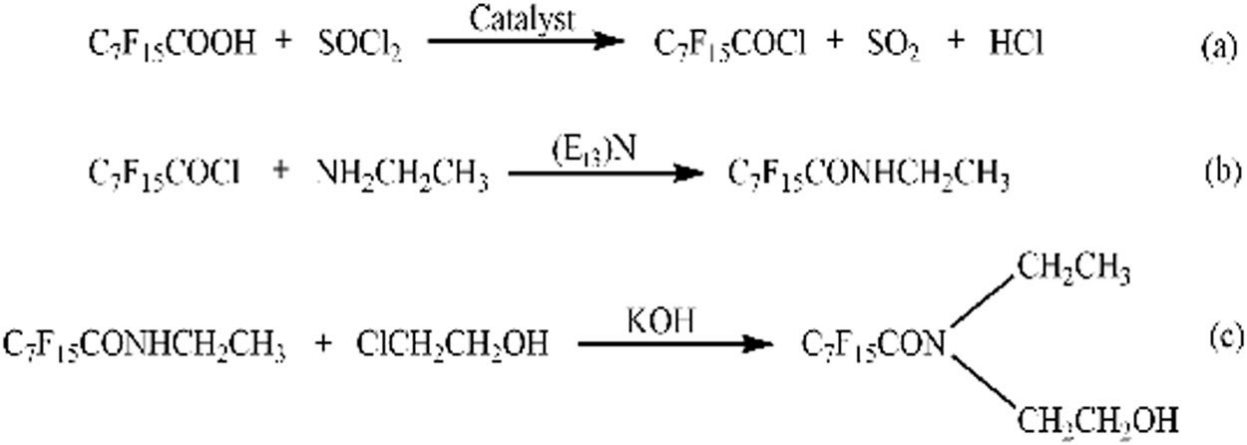
Synthesis of the novel fluorocarbon surfactant

The synthetic route of compound **3** is shown in Fig. 10 and it is carried out according to the following procedures.

First of all, perfluorooctanoic acid and catalyst were added to a glass reactor at a molar ratio of 10:1. Next, thionyl chloride (28 wt.%) was instilled at 0–5 °C. For 30 min followed by the rise of temperature reflux for 2 h. Then, it was converted into a round-bottomed flask and the 128 °C fraction was collected to give perfluorooctanoyl chloride (**1**). The first reaction is exhibited in Fig. 10(a).

Secondly, ethylamine and triethylamine in a molar ratio of 1:1 were added to a 250 ml a glass reactor with a spherical condenser with the addition of isopropyl ether as solvent. Next, the same molar ratio of **1** was instilled under a cold condition. Afterwards, the temperature was raised to 60 °C for 4 h. Then, the mixture was stirred and washed with distilled water. The organic phase transferred to a pear-shaped separatory funnel and adjusted with 0.5 wt.% hydrochloric acid to neutral. The product was then dried over anhydrous magnesium sulfate, filtered and concentrated under reduced pressure to give N-ethyl perfluorooctanoamide (**2**). The process is shown in Fig. 10(b).

At last, compound **2** and potassium hydroxide were sequentially added to a solution of anhydrous ethanol in a three-necked flask with a condenser at 40 °C for 2 h. Then, 2-chloroethanol was added dropwise to the mixture and the temperature was raised to 70 °C for 21 h. After washed with distilled water, the reaction mixture was transferred to a pear-shaped separatory funnel and the upper liquid was collected and purified under negative pressure to obtain final N-ethyl-N-hydroxyethyl perfluorooctanoamide (**3**). The reaction is exhibited in Fig. 10(c).

## Methods

### Contact angle measurement

In order to measure the contact angle of water or n-decane drops on shale surface, we used a micropipette to place droplets on the shale surface. The photo of the droplet’s topography was taken with a digital camera. The contact angle of the gas-liquid-shale three-phase was measured by the software^26^.

### Owens two-liquid method

Shale wettability is closely related to surface free energy (${\gamma }_{S}$). In order to study the wettability of shale, ${\gamma }_{S}$ was investigated by Owens two-liquid method. The detailed calculation of ${\gamma }_{S}$ is as follows^27^.

The two liquids in the formula are water and n-decane; $\,{\gamma }_{s}^{D}$ and $\,{\gamma }_{s}^{P}$ were the dispersion force and polar force, respectively; ${\theta }_{1}$ and ${\theta }_{2}$ were the contact angles of water and n-decane. Variables ${\gamma }_{S}^{D}$ and ${\gamma }_{S}^{P}$ can be calculated from Equations (3) and (4)^28^.

### Imbibition

**Figure 11:**
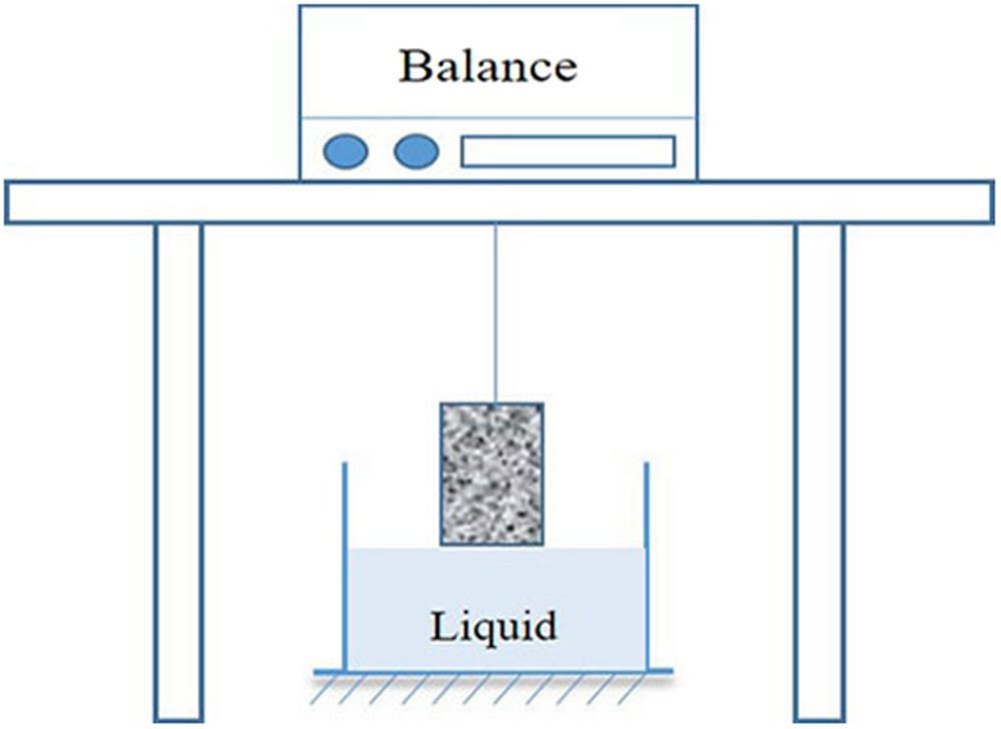
The simple diagram of imbibition experiment.

The imbibition experiment was used to further study the wettability of shale. The amount of liquid inhaled shale over time is recorded by electronic scales^29,30^. The simple diagram of imbibition experiment shown in Fig. 11.

### The glass capillary tube rise test

**Figure 12:**
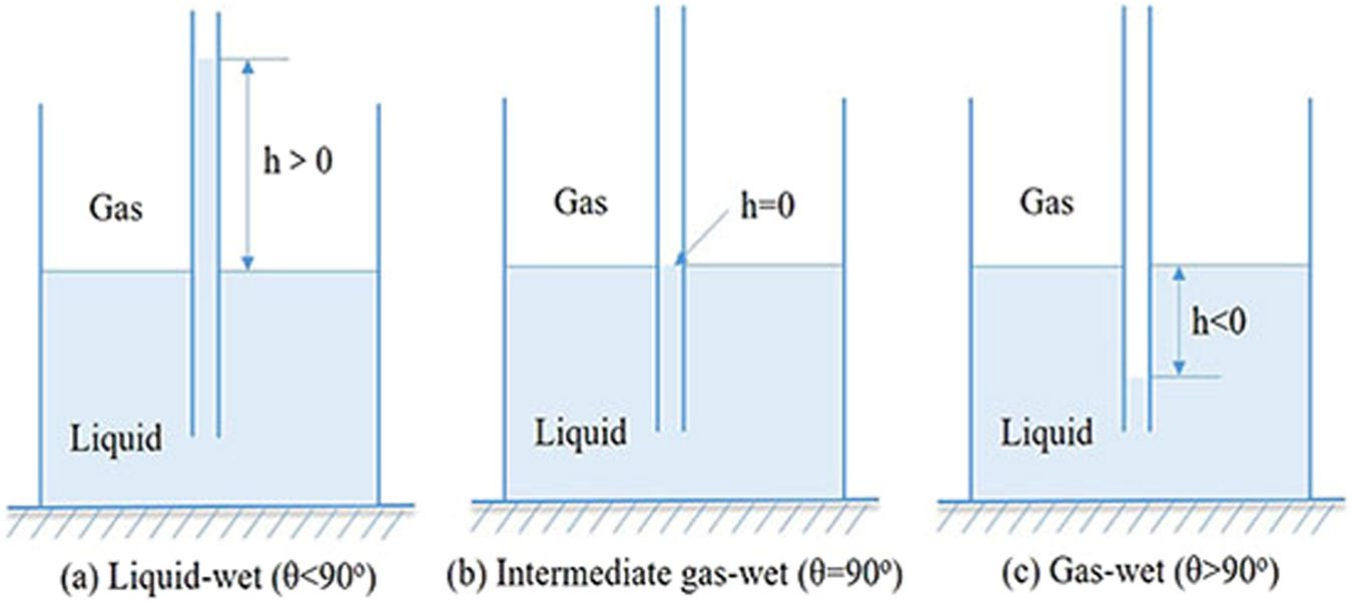
The simple diagram of capillary tube experiment.

The glass capillary tube rise experiment was performed to evaluate the effect of the novel fluorocarbon surfactant on wettability alteration of the shale surface. The diameter of the capillary used in this study is 1.0 mm. Firstly, the capillaries were cleaned and processed at high temperature. Next, the capillary was soaked with the novel fluorocarbon surfactant. Finally, the prepared capillary is inserted vertically into the liquid. The simple schematic is exhibited in Fig. 12. When the height of liquid is positive, the contact angle is less than 90°. If the height of the liquid is zero, the contact angle is 90°. When the height of the liquid is negative, the contact angle is greater than 90°. The detailed contact angle value can be calculated by Equation 5, where σ, γ and ρ are the surface tension, liquid density and capillary diameter, respectively^31,32^.

### Data availability

The datasets generated during the current study are available from the corresponding author on reasonable request.
